# RNA‐Seq identifies genes whose proteins are upregulated during syncytia development in murine C2C12 myoblasts and human BeWo trophoblasts

**DOI:** 10.14814/phy2.14671

**Published:** 2021-01-06

**Authors:** Christopher Azar, Mark C. Valentine, Julie Trausch‐Azar, Lisa Rois, Moe Mahjoub, D. Michael Nelson, Alan L. Schwartz

**Affiliations:** ^1^ Department of Pediatrics Washington University School of Medicine St. Louis MO USA; ^2^ Department of Obstetrics and Gynecology Washington University School of Medicine St. Louis MO USA; ^3^ Department of Medicine Washington University School of Medicine St. Louis MO USA; ^4^ Department of Developmental Biology Washington University School of Medicine St. Louis MO USA

**Keywords:** cell fusion, placenta, syncytialization

## Abstract

The fusion of villous cytotrophoblasts into the multinucleated syncytiotrophoblast is critical for the essential functions of the mammalian placenta. Using RNA‐Seq gene expression, quantitative protein expression, and siRNA knockdown we identified genes and their cognate proteins which are similarly upregulated in two cellular models of mammalian syncytia development (human BeWo cytotrophoblast to syncytiotrophoblast and murine C2C12 myoblast to myotube). These include DYSF, PDE4DIP, SPIRE2, NDRG1, PLEC, GPR146, HSPB8, DHCR7, and HDAC5. These findings provide avenues for further understanding of the mechanisms underlying mammalian placental syncytiotrophoblast development.

## Graphical abstract

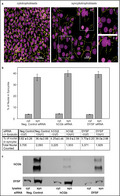

## 1 INTRODUCTION

The human placenta performs many of the diverse functions that control maternal metabolism relating to fetal growth and development that mediate the survival of the offspring. Placental dysfunction accompanies many disorders of pregnancy and underlies both short‐term and long‐term sequelae for the fetus‐newborn‐child‐adult.

Fusion of the underlying placental cytotrophoblasts forms the syncytotrophoblast which is a multinucleate continuous layer covering the large placental villous surface. Importantly, the mechanisms that govern the transition of villous cytotrophoblasts into syncytiotrophoblast (i.e., syncytium formation) are central to understanding the physiological and pathological roles of this key maternal‐fetal interface.

Syncytia formation is a highly specialized and essential process for mammalian placental development, muscle development, and formation of osteoclasts from macrophages. In addition, syncytia are a common feature of mammalian infection with enveloped viruses including lentivirus, coronavirus, paramyxovirus, and some herpes viruses. In each of these cell‐cell fusion systems, many molecules are involved to varying degrees, although the magnitude to which they participate is not always clear.

Perhaps the best‐studied mammalian system of syncytia formation is that of myoblast fusion to form multi‐nucleate myotubes and ultimately myofibers (Petrany & Millay, 2019; Schejter, 2016). Myoblast fusion is driven by two independent skeletal muscle‐specific proteins, myomaker, and myomixer/myomerger, whose expression is tightly regulated and which function at distinct steps in the fusion process (Bi et al., 2017; Millay et al., 2013). Other effectors of myoblast fusion include the actin cytoskeleton, phosphatidylserine (PS) exposure, membrane PS receptors, ferlin proteins, and the RAC1/ELMO/BAI3/Dock1 pathway (Petrany & Millay, 2019).

The fusion of placental cytotrophoblasts is tightly regulated throughout pregnancy (Simpson et al., 1992). Yet, the processes that regulate cytotrophoblast fusion with syncytotrophoblast are largely unknown. Mediators associated with cytotrophoblast fusion include syncytin‐1 and syncytin‐2 and their receptors, SLC1A5 and MFSD2A, respectively (Blaise et al., 2003; Blond et al., 2000; Bolze et al., 2017; Huppertz & Gauster, 2011; Lavillette et al., 2002; Mi et al., 2000). Syncytin‐1 and syncytin‐2 are fusogenic Env glycoproteins of human endogenous retroviral origin. Initially identified by Mi et al. (2000) and Blond et al. (2000), syncytin‐1 is a member of the HERV‐W family and supports homo‐ and heterotypic fusion when expressed in cell lines expressing the syncytin‐1 receptor, SLC1A5 (Lavillette et al., 2002). Knockout of the murine homolog of syncytin‐1 (i.e., syncytin‐A) results in the defective placental structure including excess unfused cytotrophoblasts and embryonic lethality by 13.5 dpc (Dupressoir et al., 2009). However, the presence of syncytin‐1 and its receptor in human cytotrophoblast cell lines is, in itself, insufficient for trophoblast fusion (Kudo et al., 2003). Using RNA‐Seq gene expression and quantitative protein expression we recently identified six upregulated (hCGβ, FLRG, CRIP2, TREML2, PAM, INHA) and five downregulated (SERPINF1, SAA1, C17orf96, KRT17, MMP19) genes in two cellular models of cytotrophoblast to syncytiotrophoblast development, primary human villous and human BeWo cytotrophoblasts (Azar et al., 2018).

Villous cytotrophoblasts isolated from human term placentas undergo differentiation and fusion into syncytia during culture (Azar et al., 2018; Kliman et al., 1986; Nelson et al., 1999). Moreover, the human trophoblast choriocarcinoma BeWo b30 responds to signals for fusion; we showed that cyclic AMP signaling markedly enhanced syncytia formation and hormonal differentiation (Orendi et al., 2010; Wice et al., 1990). The murine C2C12 myoblast cell line also undergoes triggered differentiation and regulated syncytia formation to myotubes in vitro (Petrany & Millay, 2019; Schejter, 2016). Herein, we exploit the power of these two cell models (BeWo, C2C12) to utilize an unbiased approach to identify genes, and their protein products, which are similarly upregulated in the two mammalian cell systems that reflect cell‐cell fusion and syncytia formation. We undertook this study with the expectation that common mechanisms or pathways would be found in myoblast fusion and cytotrophoblast fusion.

## 2 RESULTS

BeWo cytotrophoblast cells form syncytia upon forskolin treatment in vitro. Incubation with 100 µM forskolin for 72 hrs induces marked syncytia formation, as we have shown previously (Wice et al., 1990). C2C12 myoblast cells form syncytia upon differentiation in vitro. Incubation with 2% horse serum‐containing DMEM for 6d induces marked syncytia formation, as we have shown previously (Sun et al., 2005).

### 2.1 RNA‐Seq analysis of BeWo cells and C2C12 cells

**Figure 1:**
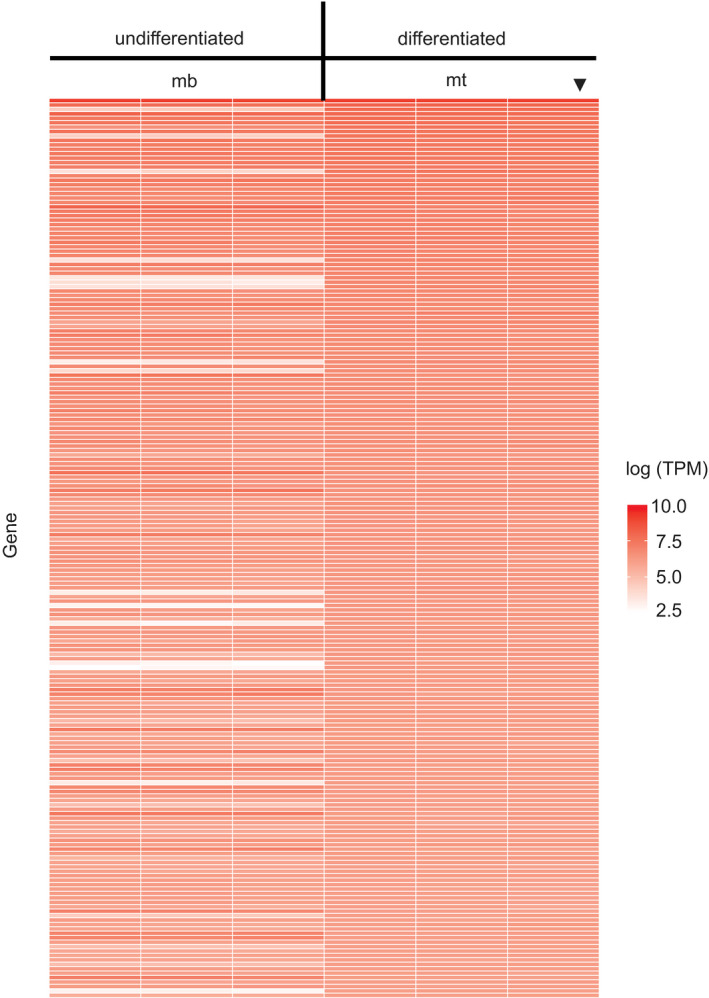
RNA‐Seq gene expression heat maps of triplicate samples for C2C12 cells at 0d (mb) and 6d (mt) in differentiation media. In the plot, the arrowhead denotes the sample used to order the heat map

**Figure 2:**
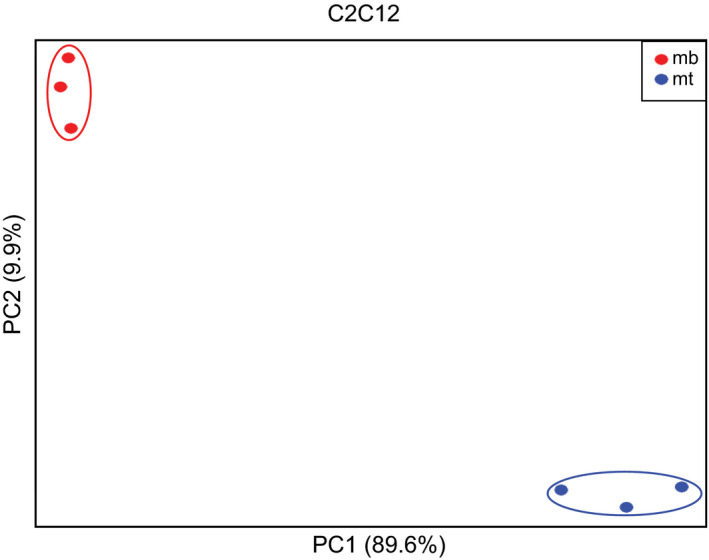
Principle components analysis of C2C12 cell gene expression data. Analysis was performed in R. The first and second principal components are plotted on the x‐ and y‐axis, respectively

RNA‐Seq was performed on triplicate preparations of BeWo cells at 0 hr and 72 hrs in the absence of forskolin (cyt) and at 24 hrs, 48 hrs, and 72 hrs (syn) in the presence of forskolin. RNA‐Seq was performed on triplicate cultures of C2C12 cells at 0d (mb) and 6d (mt) in differentiation media. There was a remarkable similarity in overall gene expression levels among the three samples at each of the time points (Figure 1 and Azar et al., 2018). There were substantial differences between the 0d and 6d C2C12 cells and between the BeWo cells ∓ forskolin at 72 hrs as shown via principle component analysis **(**Figure 2 and Azar et al., 2018). This pattern was exactly as anticipated, with minor differences between samples at the same time points in contrast with the much larger differences between time points. This captures the different transcriptional programs in place in cytotrophoblasts/syncytiotrophoblasts, and myoblasts/myotubes.

**Figure 3:**
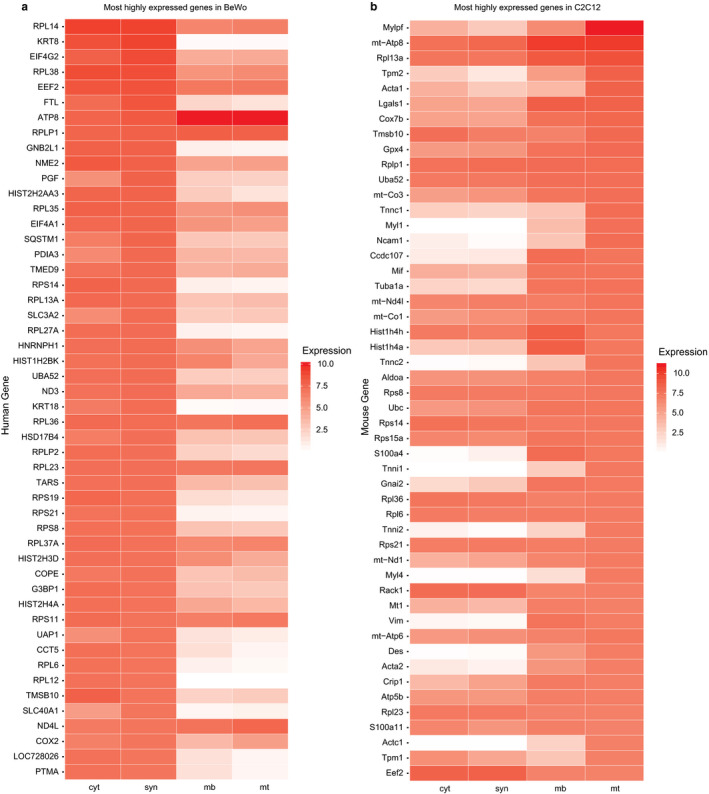
The most highly expressed genes in (a) BeWo cells and (b) C2C12 cells. In each panel, the 50 most highly expressed genes in the indicated sample were selected and used to sort the heat map. The expression levels of those genes in each of the other samples is also shown

To determine the genes differentially expressed in the 6d C2C12 myotubes (differentiated) versus the 0d C2C12 myoblasts (undifferentiated), we analyzed the 50 most upregulated genes in these two phenotypes. Not surprisingly, a number of the most upregulated genes during the C2C12 transition from myoblast to myotube were involved in muscle contractile machinery (Figure 3), whereas BeWo cells displayed numerous histone and ribosomal protein genes (Azar et al., 2018). Using PANTHER (Mi et al., 2016), we rigorously characterized the pathways and gene families in our samples. Changes in expression in C2C12 cells at 0d and 6d in differentiation media were compared with the results of those we previously reported for BeWo cells ± forskolin (Azar et al., 2018). While the gene ontology terms that were significantly enriched in the genes we analyzed in BeWo cells included sex differentiation, reproduction, tissue development, and amino acid transport, in C2C12 cells the terms most enriched in the genes we analyzed involved, perhaps not surprisingly, muscle development and muscle contraction. Other enriched pathways included cytoskeletal organization and cell cycle processes. A complete list of significant pathways is included in the PANTHER Analysis Data File.

The data from these two systems demonstrate that there are distinct similarities and differences, as expected, to the formation of syncytia in these two cellular models. Encouragingly, several of the differentially regulated genes in the myoblast to myotube transition in C2C12 cells and in the cytotrophoblast to syncytiotrophoblast transition in BeWo cells overlap. This lends support to the use of both of these models and suggests that, despite differences, both are capturing important and shared aspects of the syncytialization process.

**Figure 4:**
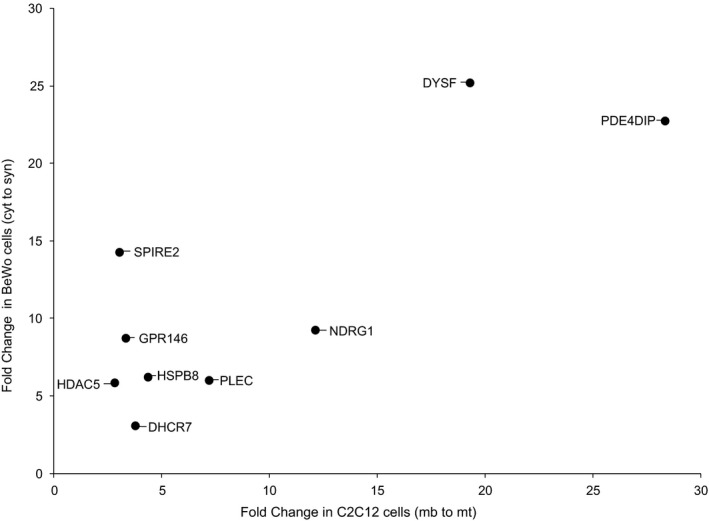
The set of genes that is differentially expressed upon fusion in both C2C12 and BeWo cells. Each gene that met our fold change and expression criteria was plotted by its fold change in C2C12 cells cultured in differentiation media from 0d (mb) to 6d (mt) on the x‐axis and the fold change in BeWo ‐forskolin (cyt) or +forskolin (syn) for 72h on the y‐axis. Only genes that were upregulated in both samples were retained

To best capture the genes that are critical for syncytialization, we combined our C2C12 differentiation and BeWo differentiation datasets into a joint analysis. To do this, we first stringently filtered each dataset independently, keeping only genes that were expressed at ≥85th percentile for at least one time point, while also showing greater than 2.5‐fold change between myoblast/myotube and cytotrophoblast/syncytiotrophoblast. We subsequently plotted the genes as points determined on their fold change in C2C12 myoblast/myotube on the x‐axis by fold change in BeWo cytotrophoblast/syncytiotrophoblast on the y‐axis (Figure 4 **)**. Viewed this way, nine genes were upregulated under both conditions and we selected these nine genes for further analysis.

### 2.2 Protein expression in C2C12 (0d vs. 6d) and BeWo (±forskolin) cells

**Figure 5:**
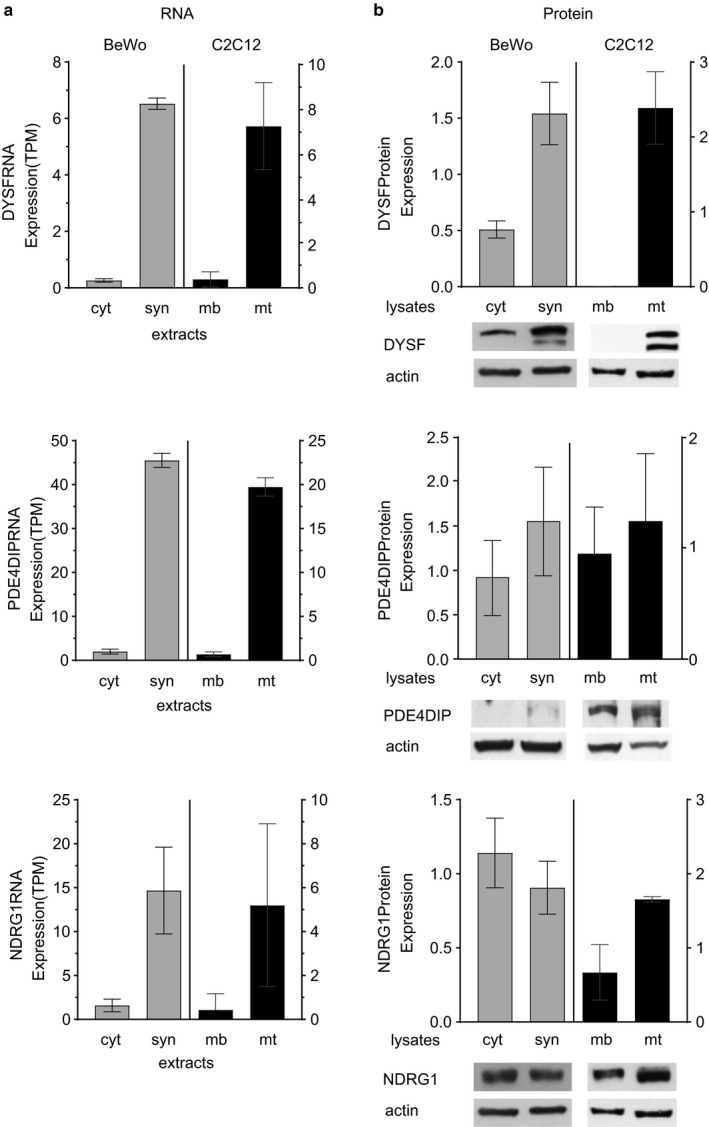
(a) RNA‐Seq analysis and (b) quantitative Western blot analysis of the three most upregulated genes of interest in BeWo cells ‐forskolin (cyt) or +forskolin (syn) and C2C12 cells in differentiation media at 0d (mb) or 6d (mt). RNA‐Seq results are expressed in transcripts per million (TPM). Immunoblots were quantified and normalized to levels of actin; note, multiple panels in Figures 5 and 6 used the same actin control

**Figure 6:**
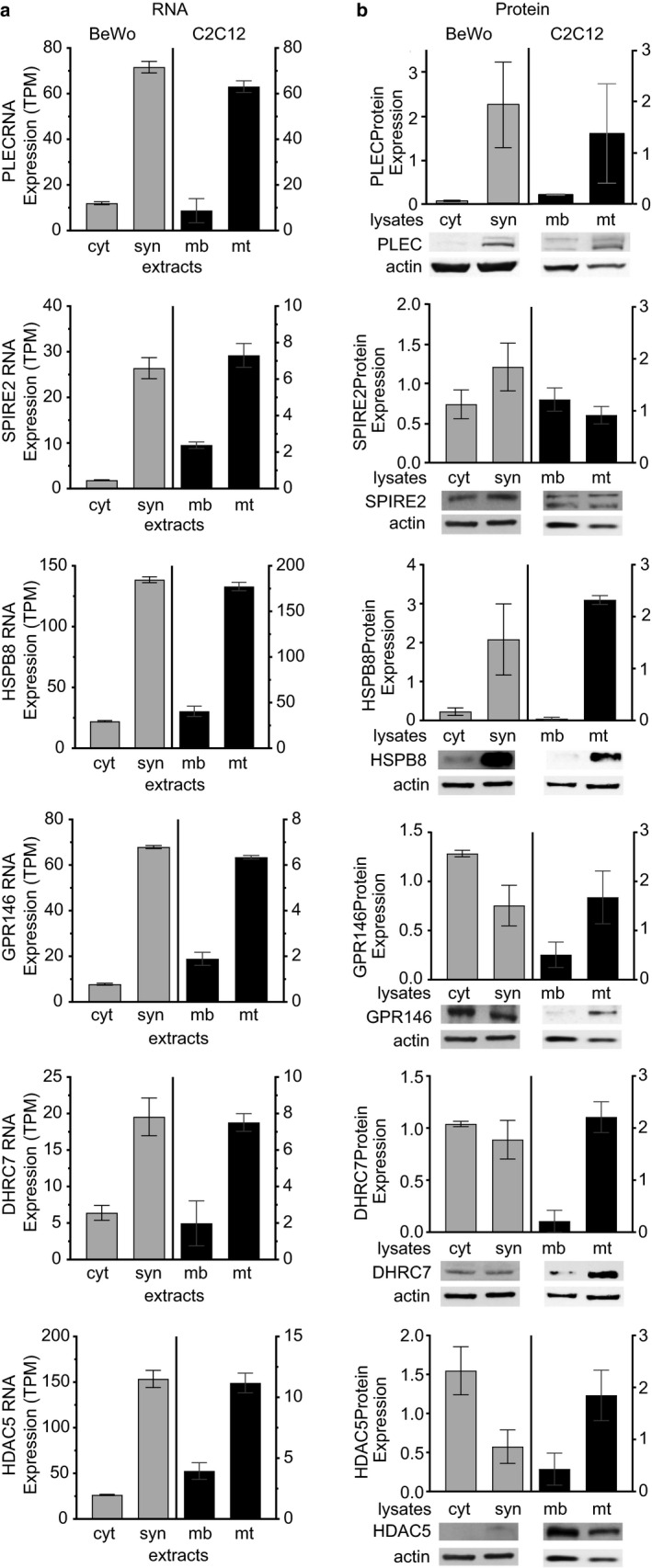
(a) RNA‐Seq analysis and (b) quantitative Western blot analysis of the six remaining upregulated genes of interest in BeWo cells –forskolin (cyt) or + forskolin (syn) and C2C12 cells in differentiation media at 0d (mb) or 6d (mt). RNA‐Seq results are expressed in transcripts per million (TPM). Immunoblots were quantified and normalized to levels of actin; note, multiple panels in Figures 5 and 6 used the same actin control

In order to determine if the changes in RNA levels (i.e., RNA‐Seq expression) in C2C12 (0d vs. 6d) and BeWo (±forskolin) are reflected in the levels of the respective proteins, we performed quantitative Western blot analysis of C2C12 cell lysates at 0d and 6d and BeWo cell lysates at 72 hrs ±forskolin. We selected individual antibodies that recognize both the human and murine proteins. Table 1 lists the specific antibodies used for these analyses. As seen in Figures 5, 6, and Table 2, for the nine genes which are upregulated, the increase in protein expression in cell lysates generally correlated with the increase in RNA expression.

**Table 1:** Description of antibodies used for immunoblots, immunohistochemistry, and immunofluorescence

Protein	MW kDa	Company	Antibody	Host	Lot #
DYSF	237	Abcam	ab124684	Rabbit m	GR211867‐33
PDE4DIP	265	Sigma	HPA008162	Rabbit p	A89686
SPIRE2	80	Invitrogen	PA5‐24099	Rabbit p	UC2732201A
NDRG1	43	Biorbyt	orb381904	Rabbit p	QA2100
PLEC	523	NSJ Bio	R32233	Rabbit p	R32233‐413052
GPR146	37	Invitrogen	PA5‐27175	Rabbit p	UE2775116A
HSPB8	22	Invitrogen	PA5‐76780	Rabbit p	UC2732613A
DHCR7	54	Abcam	ab103296	Rabbit p	GR3225939‐4
HDAC5	122	Abcam	ab55403	Rabbit p	GR36969‐13
actin	42	Sigma	A5441	Mouse m	064M4789V
MHC	200	Santa Cruz	sc‐376157	Mouse m	A0819
hCGβ	18	Abcam	ab3976	Rabbit p	GR254346‐B
ZO‐1	220	Invotrogen	40–2200	Rabbit p	UB280595

Note

In host column p = polyclonal, m = monoclonal.

**Table 2:** Summary of expression data from RNA‐Seq and quantified immunoblot results shown in Figures 5, 6

Gene	BeWo Expression						C2C12 Expression					
	RNA			Protein			RNA			Protein		
	cyt	syn	Fold Change	cyt	syn	Fold Change	mb	mt	Fold Change	mb	mt	Fold Change
DYSF	0.26	6.6	25	0.51	1.5	2.9	0.38	7.3	19	0.003	2.4	>100
PDE4DIP	2.0	46	23	0.91	1.5	1.6	0.70	20	29	0.94	1.2	1.3
NDRG1	1.6	15	9.4	1.1	0.91	0.83	0.43	5.2	12	0.67	1.7	2.5
PLEC	12	72	6.0	0.05	2.2	44	9	63	7.0	0.19	1.4	7.4
SPIRE2	1.9	26	14	0.75	1.2	1.6	2.4	7.3	3.0	1.2	0.92	0.77
HSPB8	22	139	6.3	0.23	2.1	9.1	41	177	4.3	0.03	2.3	77
GPR146	7.8	68	8.7	1.3	0.75	0.58	1.9	6.3	3.3	0.51	1.7	3.3
DHCR7	6.4	20	3.1	1.0	0.89	0.89	2.0	7.5	3.8	0.21	2.2	10
HDAC5	26	154	5.9	1.5	0.57	0.38	3.9	11	2.8	0.43	1.8	4.2

Note

Data for BeWo cells –forskolin (cyt) or + forskolin (syn) and C2C12 cells at 0d (mb) or 6d (mt) in differentiation media are shown. Increases or decreases in expression are given as fold change.

### 2.3 Effect of siRNA knockdown of hCGβ and dysferlin in BeWo cells

**Figure 7:**
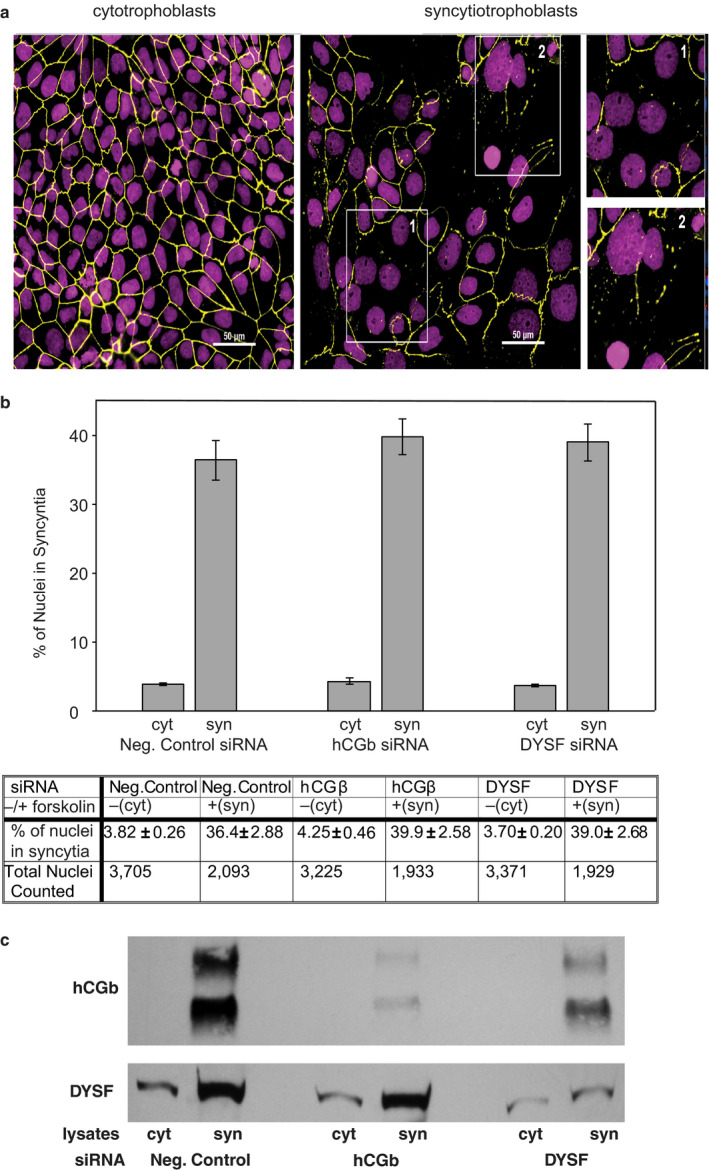
Effect of siRNA knockdown of hCGβ or DYSF expression on the syncytialization of BeWo cells. (a) Representative double‐label immunofluorescence of BeWo cytotrophoblasts (left) and syncytiotrophoblasts (right). BeWo cells ‐forskolin (cyt) or +forskolin (syn), were fixed, and nuclei were stained with DAPI (shown in magenta), while cell junctions were stained with anti‐ZO1 antibody (pseudo‐colored in yellow). Insets of syncyntia shown on the right were magnified 2‐fold. (b) Results of nuclei counts used to determine percent syncytialization and (c) representative Western blots of BeWo cells transfected with siRNA targeting the genes indicated (hCGβ and DYSF) and treated with either vehicle (cyt) or forskolin (syn) for 72h as described in Materials and Methods

As dysferlin was the most upregulated gene in both BeWo and C2C12 cells (Figure 4), and hCGβ is the most upregulated gene in BeWo cells (Azar et al., 2018) we next examined the effects of hCGβ knockdown and dysferlin knockdown in BeWo cells treated with forskolin for 72 hrs. As seen in Figure 7, knockdown of hCGβ markedly (>95%) reduced cellular hCGβ levels in cells treated with forskolin. Surprisingly, cellular dysferlin levels were also markedly reduced. Knockdown of dysferlin also markedly (>95%) reduced cellular dysferlin levels in cells treated with forskolin, whereas hCGβ levels were largely preserved. Under both conditions (i.e., hCGβ knockdown or dysferlin knockdown), however, BeWo cell fusion was unaffected (Figure 7). (*p* > .05 control vs. hCGβ knockdown vs. dysferlin knockdown).

## 3 DISCUSSION

Syncytia formation is a highly specialized and indispensable process for mammalian placental development and muscle development. The data herein show a comprehensive, unbiased RNA‐Seq screen that identified genes that likely play a role in syncytia development in placental cytotrophoblast to syncytiotrophoblasts and in muscle myoblast to myotubes. The RNA‐Seq analysis was performed on samples from two complementary and biologically relevant in vitro model systems of syncytialization: forskolin‐induced differentiation of cytotrophoblasts to syncytiotrophoblasts in the human choriocarcinoma‐derived cell line, BeWo and growth factor depletion‐induced differentiation of myoblasts to myotubes in the murine skeletal muscle‐derived cell line, C2C12. We selected these as among the best characterized mammalian cell systems, recognizing one is human (BeWo), the other murine (C2C12). One potential benefit thereof is the additional selection criterion of the conservation of genes/proteins across species. We used stringent criteria to identify the most highly upregulated genes in the two model systems interrogated from the RNA‐Seq data. We identified nine genes from this analysis and pursued the evaluation of protein expression of each of these in the BeWo and C2C12 systems via quantitative Western blot analysis. The data show that the RNA and protein expression levels do not correlate uniformly.

Many of the nine upregulated genes encode proteins involved in cytoskeletal dynamics, while others regulate cellular functions. Mutants of many are associated with muscle disorders. Dysferlin (DYSF, ~237kDa) is strongly expressed in skeletal muscle and placenta in addition to heart and brain(Liu et al., 1998). In mature skeletal muscle, dysferlin localizes to the plasma membrane, whereas in C2C12 myotubes it is found in T‐tubules but translocates to the plasma membrane upon damage. Dysferlin has numerous binding partners, including caveolin‐3/MG53 as well as AHNAK/annexin/actin complexes (Lek et al., 2012). Dysferlin is mutated in Miyoshi myopathy and limb‐girdle muscular dystrophy (Bansal et al., 2003; Liu et al., 1998). Robinson et al. (2009), demonstrated dysferlin expression in the apical plasma membrane of human placental syncytiotrophoblast and increased dysferlin expression in BeWo cells treated with forskolin, suggesting a relationship between cytotrophoblast fusion and dysferlin expression. Furthermore, dysferlin knockout mice are viable, fertile, and have a normal growth rate (Bansal et al., 2003). Phosphodiesterase 4D interacting protein, (PDE4DIP, also known as myomegalin, ~260 kDa) anchors PDE4 to the centrosome‐Golgi region and is in sarcomeres of skeletal muscle and heart (Verde et al., 2001). Knockout of PDE4DIP’s DUF1220 domain reduced fecundity to ~68% of predicted offspring (Filleur et al., 2009). Spire type actin nucleation factor 2 (SPIRE2, 80 kDa), a cytoplasmic actin nucleation factor, provides a link between actin cytoskeletal dynamics and intracellular transport (Schuh, 2011). N‐myc downstream‐regulated gene 1 (NDRG1, 43kDa) is the most ubiquitous member of the NDRG family, expressed in the cytoplasm and nucleus and the only family member expressed in placenta. We previously showed that NDRG1 attenuates hypoxic injury to human trophoblasts (Chen et al., 2006). Expression of NDRG1 (also termed RTP) is markedly increased in BeWo cells following forskolin treatment (Xu et al., 1999). In addition, mutations in NDRG1 are associated with Charcot‐Marie‐Tooth disease type 4D. NDRG1 knockout mice were born with the normally expected Mendelian frequency, were viable, and were fertile (Okuda et al., 2004). Plectin (PLEC, ~500kDa), a cytoplasmic protein, is widely expressed in muscle, placenta, and heart where it interacts with cytoskeletal components including actin, microtubules, intermediate filaments, and the plasma membrane (Rezniczek et al., 2016). Plectin knockout mice are viable but die at 2–3 days and exhibit marked skin abnormalities. GPR146 (37 kDa), an orphan G protein‐coupled receptor, has been proposed as a C‐peptide receptor. Most recently, Yu et al. (2019) showed that GPR146 regulates plasma cholesterol levels via ERK signaling and that GPR146 knockout mice are viable and fertile. Heat shock protein HSPB8 also known as HSP22 (22 kDa) is a cytoplasmic protein that serves as a member of the chaperone‐assisted autophagy complex and is involved in Charcot‐Marie‐Tooth disease type 2L as well as in a rare form of human distal myopathy (Ghaoui et al., 2016). HSPB8 knockout mice are viable (Qiu et al., 2011). 7‐dehydrocholesterol reductase (DHCR7, 54 kDa) catalyzes the final step in cholesterol biosynthesis, the conversion of dehydrocholesterol to cholesterol, primarily within the endoplasmic reticulum of virtually all tissues. Mutations in DHCR7 are associated with Smith‐Lemli‐Opitz Syndrome (Wassif et al., 1998) and null variants are viable as a result of maternal to fetal cholesterol transport. Histone deacetylase 5 (HDAC5, 122 kDa) deacetylates target lysine residues of core histones and thus provides epigenetic regulation of numerous developmental events including suppressing transcription of MEF2C during muscle differentiation (McKinsey et al., 2000). HDAC5 knockout mice are viable and fertile (Chang et al., 2004).

The fusion of villous cytotrophoblasts throughout pregnancy generates syncytiotrophoblast with a ~10M^2^ surface area (Bolze et al., 2017). While villous cytotrophoblast to syncytiotrophoblast fusion requires syncytin‐1 and syncytin‐2 and their ubiquitous receptors, membrane transporters SLC1A5 and MFSD2A in humans, many additional factors including PS exposure and annexin A5 are required for cytotrophoblast fusion (Degrelle et al., 2017; Huppertz & Gauster, 2011).

The fusion of skeletal muscle myoblasts to form multinucleated myotubes and ultimately myofibers occurs throughout the lifetime of the muscle and allows regeneration and adaptation to exercise. Myomaker and myomixer/myomerger are essential for vertebrate muscle formation. Myomaker/myomixer expression and activity are highly regulated to the time and place of myoblast fusion during development. One current stepwise model supports myomaker as necessary for hemifusion whereas myomixer mediates pore formation (Petrany & Millay, 2019). When co‐expressed, myomaker and myomixer drive fibroblast fusion, indicating that the combination of the two proteins can perform the steps necessary for fusion. However, other factors identified may act at different stages of the process and include actin cytoskeletal remodeling, PS exposure, BAI1 and stabilin 2 membrane PS receptors, VASp/Stlr/Arp2/3, annexins, cadherins, ferlin proteins, the RAC1/ELMO/BAI3/Dock 1 pathway (Petrany & Millay, 2019).

**Figure 8:**
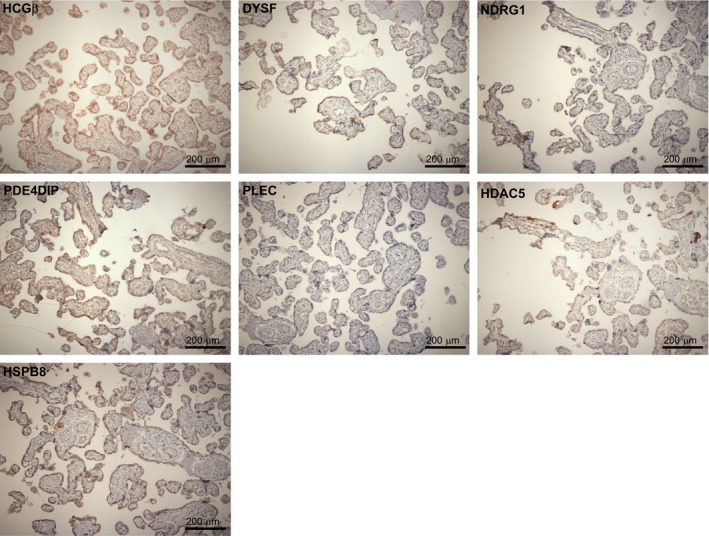
Immunochemical staining of normal term human placental villi. Serial sections of normal term human placental villi samples were immunostained with antibodies to upregulated proteins and hematoxylin & eosin, as indicated. Robust HRP staining of syncytiotrophoblasts is clearly visible for upregulated proteins

For our analysis, we selected antibodies that recognized both human and murine protein species of our nine candidate genes. Of note, all of these nine proteins are enriched in syncytiotrophoblast surrounding the villi of normal human placenta (Figure 8). Taken together, we conclude that the activities of these nine similarly regulated gene products do not appear to behave as a single uniform function during the transition from mononucleated cytotrophoblast to differentiated syncytiotrophoblast and from myoblast to myotube, and likely upregulation of some is required for fusion, while upregulation of others is a result of fusion, or unrelated to the fusion process per se. Indeed, future analyses of serum‐depletion or forskolin treatment of myomaker‐deficient (fusion incompetent) C2C12 cells may provide insight into this.

Gene expression changes have been examined in BeWo cells (Kudo et al., 2004; Shankar et al., 2015; Zheng et al., 2017) during forskolin treatment. Kudo et al. (2004) used microarray and RNA‐Seq analysis to track up or downregulation of gene expression and kinetic changes during the trophoblast formation of syncytium. Zheng et al. (2017) performed RNA‐Seq yielding data for BeWo cells (of the parent choriocarcinoma cell line) to identify and cluster differentially expressed genes. Shankar et al. (2015) augmented their RNA‐Seq data with DNA methylation and ChIP‐seq assays to generate a more complete epigenetic profile of BeWo cytotrophoblasts and syncytiotrophoblasts.

Several previous studies have explored the transcriptional changes that occur during the transition from myoblasts to myotubes in C2C12 cells. Moran et al. (2002) used microarrays to interrogate gene expression levels in these cells at several timepoints during differentiation. They used this time and expression data to cluster genes into groups based on their expression profile. They found that these clusters of genes were enriched for genes involved in cell‐cycle regulation, muscle contraction, cytoskeletal organization, and cellular metabolism. Our results herein are consistent with those of Moran et al. (2002); that is, many of the same functional classes were found to be overrepresented in both studies. More recently, Trapnell et al. (2010) used RNA‐Seq to explore the transcriptional changes in C2C12 myoblasts and myotubes. While the primary goal of that work was to detect novel transcripts and unannotated isoforms, the differentially expressed and differentially spliced genes that they discuss are again consistent with our findings. Several other papers have explored the role of MyoD1, PPARɣ, and other transcription factors critical for the differentiation process (He et al., 2017; Shintaku et al., 2016). These also show that the transcriptional changes orchestrated by these transcription factors affect the same genes that we found to be differentially regulated. In contrast to previous works, our current study explicitly looks at the process of syncytialization. This is one necessary step in the formation of myotubes that has not been well‐characterized. By comparing the differentially expressed transcripts between myoblasts and myotubes with the differentially expressed transcripts between cytotrophoblasts and syncytiotrophoblasts, we are able to identify those transcripts that may play a direct role in the syncytialization process.

The most upregulated gene and protein expression during placental development is hCGβ. Yet, the literature is inconsistent in the relationship between cytotrophoblast fusion and hCGβ expression. Saryu Malholtra et al. (2015) have suggested that the knockdown of hCGβ in BeWo cells is associated with reduced forskolin‐induced cell fusion. Dysferlin was the most upregulated gene and protein expression in both C2C12 and BeWo cells as described above (Figure 4). Robinson et al. (2009) have shown that dysferlin is expressed primarily in syncytiotrophoblast in the human placenta and that expression of dysferlin correlated with forskolin‐induced fusion of BeWo cytotrophoblasts. Following dysferlin knockdown, Omata et al. (2013) have reported BeWo cell fusion is unaltered. Our results show that following the knockdown of either hCGβ or dysferlin in BeWo cells, forskolin‐induced syncytia formation (i.e., cell fusion) was unaltered (Figure 7). Thus, these results argue that neither hCGβ nor dysferlin upregulation is essential for syncytia formation.

Ultimately, for both muscle and placental syncytia formation programs, it will be essential to understand whether (a) each factor upregulates positive effectors, downregulates negative effectors or post translationally regulates essential effectors, as well as whether (b) each factor is a true membrane‐active driver of fusion, a fusion‐assisting molecule or gene product that is upregulated as a result of fusion that may be required for the proper function of the syncytia (Brukman et al., 2019; Petrany & Millay, 2019).

## 4 MATERIALS AND METHODS

### 4.1 Cells and culture

BeWo b30 cells were cultured in F‐12K Nutrient Mix, Kaighn's Modification (Gibco), 10% FBS, and 2mM glutamine (Wice et al., 1990). At 10% confluency, media was replaced with fresh media containing either 1% DMSO (control) or 100 µM forskolin in DMSO at 0 hr, 24 hrs, or 48 hrs as indicated. At the appropriate time, cells, media or both were harvested for RNA‐Seq, protein analysis or immunofluorescence as indicated.

C2C12 mouse myoblast cells were obtained from ATCC and routinely propagated in growth media (Dulbecco's Modified Eagle's Medium (DMEM) (Sigma) supplemented with 10% FBS). Myogenic differentiation to myotubes was induced by changing to differentiation media (DMEM supplemented with 2% horse serum) when the cells reached confluence. Cells were maintained in differentiation medium for 6 days with medium changed every 24 hrs (Sun et al., 2005). At the appropriate time (0d for myoblasts, 6d for myotubes) cells were harvested for RNA‐Seq, protein analysis or immunofluorescence as indicated.

### 4.2 Protein analysis

**Figure 9:**
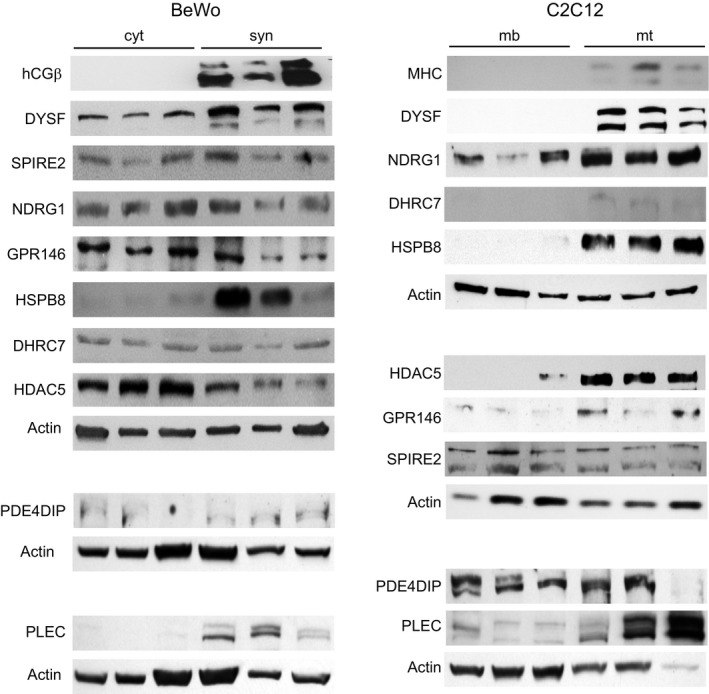
Triplicate Western blots of upregulated genes of interest in BeWo cells ‐forskolin (cyt) or +forskolin (syn) and C2C12 cells in differentiation media for 0d (mb) or 6d (mt). Blots are grouped with the actin blot that was used for their normalization. hCGb and myosin heavy chain (MHC) are included as markers of syncytialization in BeWo cells and differentiation in C2C12 cells, respectively

For cellular protein analysis, BeWo or C2C12 cells were rinsed in PBS at 4°C and thereafter incubated for 20 min at 4°C with fresh lysis buffer, followed by centrifugation (15 min at 13,000 rpm at 4°C), and the resultant supernatant was stored at −20°C, as described previously (Trausch‐Azar et al., 2015). Western blot analysis following SDS‐PAGE was performed on aliquots and probed with antibodies as described in the text and Table 1. Actin content was evaluated on each set of lysates as a control and for normalization, as indicated in the respective Figures 5, 6, and 9. Myosin heavy chain and hCGβ expression served as differentiation markers for myotubes and syncytiotrophoblasts, respectively. Western blots were developed with Pierce ECL Western Blotting Substrate, SuperSignal West Femto Maximum Sensitivity Substrate from Thermo Scientific or Bio‐Rad Clarity Western Blot ECL Substrate. Western Blots were quantitated using Image J software (https://imagej.nih.gov/ij/docs/faqs.html) and results were normalized to actin. Analyses were performed on at least three independent preparations of BeWo and C2C12 cells (see Figures 5, 6, and 9).

### 4.3 siRNA knockdown analysis

BeWo cells were transfected using nucleofection, an electroporation‐based system. Cells were cultured as described above in 10cm dishes until 80%–90% confluent then trypsinized and counted. 10 × 10^6^ cells/ml were resuspended in cell line solution V and 1 × 10^6^ cells were transfected with 10 μl of a 20 μM solution of the indicated siRNA (Santa Cruz Biotechnology, USA) following the manufacturer's instructions using program A‐020 (Lonza, Switzerland). Following transfection, cells were plated in 6 well plates. After 24 hr, 72 hrs forskolin treatment was begun and cells were harvested at indicated times as described above. Transfections and time courses were performed in duplicate in each experiment.

### 4.4 Immunofluorescence analysis

For immunofluorescence analysis and as described previously (Azar et al., 2018; Trausch‐Azar et al., 2015; Wice et al., 1990). BeWo cells were seeded onto glass coverslips in six‐well dishes and treated with forskolin or DMSO as described above. Cells were then washed with PBSc, fixed with 4% paraformaldehyde, and permeabilized in 1% Triton‐X 100. After blocking in 1% BSA/PBSc, coverslips were immunostained with primary antibody to determine subcellular localization. After washing with PBSc, coverslips were incubated with the appropriate Alexa Fluor 488 or 568 secondary antibodies (Invitrogen/Molecular Probes) and DAPI and mounted using Mowiol containing 2.5% 1, 4‐diazo‐bicyclo‐[2.2.2]‐octane (DABCO, Sigma).

Images of fixed cells were captured using a Nikon Eclipse Ti‐E inverted confocal microscope equipped with a 20X air objective lens (Nikon, Melville, NY). A series of digital optical fields (4 × 4) were captured using an Andor Neo‐Zyla CMOS camera at room temperature. Optical fields were stitched together and reconstructed using the Nikon Elements AR 4 Software.

### 4.5 Syncytialization quantitation

In immunofluorescence images, a structure was considered a syncytium if multiple nuclei were surrounded by a single cell membrane. Syncytialization was quantified by counting the total number of nuclei and the number of nuclei in syncytia in five images of each of the six conditions shown in Figure 7. Data for each condition were combined and percentages of nuclei in syncytia were calculated.

### 4.6 Statistical analysis

Statistical analysis, *SEM*, and *t*‐test comparisons were performed using ANOVA software. Significance is *p* < .05.

### 4.7 RNA‐Seq

Total RNA was isolated from BeWo and C2C12 cells using the RNeasy Plus Mini Kit (Qiagen) according to the manufacturer's instructions. QIAshredder was used for homogenization. The amount of total RNA extracted was quantified on a NanoDrop 2000 spectrophotometer and RNA quality and purity were further evaluated using an Agilent 2100 Bioanalyzer (Agilent Technologies, Santa Clara, CA, US). RNA‐Seq was performed at the Genome Technology Access Center in the Department of Genetics at Washington University School of Medicine (Azar et al., 2018). Briefly, ribo‐depleted libraries were prepared from the RNA, and samples were indexed and sequenced on an Illumina HiSeq 2500. Sequencing results from this study have been deposited in the NCBI Short Read Archive under accession number PRJNA397241 and PRJNA54661. Raw reads were pseudo‐aligned using kallisto version 0.42.4 using options “—single –b 10 –l 220 –s 20” (Bray et al., 2016).

### 4.8 Bioinformatic analysis

All analyses were performed in R version 3.2.1. Transcript abundance was determined by sleuth (Pimentel et al., 2017) and measured in transcripts per million (TPM). To determine which transcripts were differentially expressed, we applied a filter requiring a transcript to be expressed at or above the 85th percentile in at least one sample and have a 2.5‐fold change in expression between syncytiotrophoblast samples and cytotrophoblast samples and likewise between myoblast samples and myotube samples. Differential gene expression was determined by comparing C2C12 (0d) (mb) to C2C12 (6d) (mt) using the EdgeR package in R(Robinson et al., 2010) (EdgeR Data File) and the CuffLinks package (Trapnell et al., 2012, 2013) (CuffLinks Data File). To generate the heat maps, one sample was designated as the index and used to sort the transcripts. The transcript levels for every other sample were ordered according to this index sample and displayed as log (TPM).

Gene list enrichment was performed using the PANTHER (Mi et al., 2016) online suite of tools. We provided a list of the most significant genes as input. The resulting gene ontology categories were reported. The default setting of the PANTHER analysis is to perform a Bonferroni correction for multiple test correction. Thus, the reported results are unlikely to be false positives.

## CONFLICT OF INTEREST

None of the authors has any financial conflict or conflict of interest.

## AUTHOR CONTRIBUTIONS

ALS, DMN, CA and JST‐A conceived of the study. CA, MV, MM, JST‐A performed the analyses. CA, MV, JST‐A, and ALS wrote manuscript.

## DATA FILES

PANTHER Pathway analysis of differentially expressed genes in C2C12 cells in differentiation media at 0d and 6d. The 250 most significantly differentially expressed (DE) genes were provided as inputs to PANTHER. The Gene Ontology biological processes that were enriched for our list of differentially expressed genes are listed.

EdgeR analysis of differentially expressed genes in C2C12 cells in differentiation media at 0d (mb) and 6d (mt). The genes that were significantly differentially expressed (FDR <= 0.05) are included.

CuffLinks analysis of differentially expressed genes in C2C12 cells in differentiation media at 0d (mb) and 6d (mt). The genes that were significantly differentially expressed (q‐value <= 0.05) are included.

## Data Availability

The sequencing results from this study have been deposited in the NCBI Short Read Archive under accession number PRJNA397241 and PRJNA543661.
